# Improving Inpatient Flow in Adult Mental Health Services: A Structured Peer-Supported Model to Reduce Length of Stay

**DOI:** 10.1192/bjo.2026.11466

**Published:** 2026-06-30

**Authors:** Liji Premlal Puliparambil, Joanne Roberts, Amrith Shetty, Raghu Saligrama, Holly Higgs

**Affiliations:** Cheshire & Wirral Partnership NHS Foundation Trust, Chester, United Kingdom

## Abstract

**Aims:**

This quality improvement project (QIP) aimed to evaluate the impact of a structured escalation framework on identifying, reviewing, and managing patients with prolonged length of stay (LOS) of more than or equal to 30 days in adult mental health inpatient settings from 1 August 2025 to 31 January 2026 in Cheshire & Wirral Partnership NHS Foundation Trust which has six acute inpatient wards where patients are either admitted informally or detained on mental health law.

This project was led by the Rehabilitation access team of Mental health Intensive Support team (MhIST) following the Trust policy to identify the escalation process for the prolonged length of stay. The policy had identified to trigger predefined LOS thresholds: ≥30, ≥40, ≥50, and ≥60 days. It was identified that patients stay due to extended period for clinical optimisation and red alert where they are clinically ready for discharge, but barriers identified internally through the clinical community teams or externally through Local authority. For all external delays, the cases were further escalated through Multiagency Discharge Escalation (MADE) meetings for the suitable pathway.

These barriers cause increased waiting time for admission from Accident & Emergency services leading to delay for appropriate treatment to be initiated and out of area admissions for acute care treatment that takes these patients away from their family and clinical team.

**Methods:**

A Board Round framework incorporating the LOS thresholds in the weekly multidisciplinary in-reach meetings was arranged as an interface between inpatient and rehabilitation team. Key clinical and operational questions were embedded to support goal-directed care planning. Data were collected by designated care navigators, and followed by our team including LOS, reasons for delay, escalation actions, and outcomes.

**Results:**

Prior to this prolonged LOS weekly in-reach meeting, the average length of stay days in August 2025 was 74.3 and after the initiation of this meeting, it reduced to 54.2. It also showed the closure of all 29 out of area contracted acute beds. Out of a total of 548 patients, 39.5% (217) were identified as prolonged LOS. Seventy-eight percent (171) were due to clinical factors and 21.1% (46) were due to external delays. Majority (86.6%, 188) suffered with severe mental health illness. There was female predominance noted in both cohorts of delay.

**Conclusion:**

This QIP has helped to identify and reduce the barriers in the discharge pathway, thus improving the inpatient flow. The continued multidisciplinary wider team involvement was the most effective intervention to help work collaboratively.

